# A Case of Serotonin Syndrome Precipitated by Quetiapine in a Middle-Aged Female on Trazodone and Sertraline

**DOI:** 10.7759/cureus.27668

**Published:** 2022-08-04

**Authors:** Samyukta Varma, Sona Xavier, Saral Desai, Syed Ali

**Affiliations:** 1 Internal Medicine, Madurai Medical College, Madurai, IND; 2 Psychiatry, Jackson Park Hospital, Chicago, USA; 3 Psychiatry, Tower Health/Phoenixville Hospital, Phoenixville, USA; 4 Psychiatry, Drexel University College of Medicine, Philadelphia, USA; 5 Internal Medicine, Jackson Park Hospital, Chicago, USA

**Keywords:** complications, management, diagnosis, trazodone, quetiapine, serotonin syndrome (ss)

## Abstract

Serotonin syndrome (SS) is a potentially life-threatening condition caused by drugs that act on serotonergic receptors or alter serotonin metabolism. We present a case of SS in a middle-aged female who was taking trazodone and sertraline as her home medications and developed SS after being started on quetiapine during her hospital course. A 54-year-old female with a past medical history of dementia and bipolar disorder was brought to the emergency department from a nursing home for altered mental status. Delirium was ruled out. Initial blood work was significant for an elevated creatine phosphokinase (CPK) level of 753 U/L. She was started on Quetiapine 100 mg bis in die (BID) after admission as she had a history of bipolar disorder and she was having acute mood symptoms (impulsive, irritable, confrontational, belligerent, and unable to be redirected). On the second day of admission, the patient started having diaphoresis, tremors, hyperreflexia, myoclonus, and ocular clonus. A diagnosis of SS was made using Hunter’s criteria. All serotonergic medications were discontinued after which the patient started improving. She was also started on supportive therapy including IV fluids, lorazepam, and cyproheptadine. The patient was discharged on the fourth day of admission.

## Introduction

Serotonin syndrome (SS), also known as serotonin toxicity, is a potentially severe drug-induced toxidrome that can be life-threatening [1-2]. Drugs that can alter serotonin metabolism or act as an agonist on serotonin receptors cause SS. The most common drugs that cause it are selective serotonin reuptake inhibitors, monoamine oxidase inhibitors, and serotonin-norepinephrine reuptake inhibitors [3]. Prescription drug use, intentional drug overdose, and unintended drug interactions result in serotonergic hyperactivity in both the central and peripheral nervous systems. Three attributes of SS are key to understanding the disorder. First, it is an avoidable syndrome and not an idiopathic reaction. Second, the excess serotonin can manifest as a spectrum of findings -- neuromuscular abnormalities, autonomic hyperactivity, and altered mental status. Third, the clinical features can range from being barely detectable to lethal [2]. Though fatal, it can be potentially treated.

The Hunter diagnostic criteria are used to make a diagnosis of SS. This criterion is predominantly used for moderate to severe cases. Only tremor along with hyperreflexia is considered mild symptom in this criterion. However, previous literature suggests that other mild symptoms could be present in patients like anxiety, diaphoresis, tachycardia, restlessness, and shivering [3-5]. It is important to be aware of the mild symptoms too, as either self-poisoning or when a pro-serotonergic drug is added can both lead to a pronounced clinical deterioration in a short time [6]. In this case study, we describe a case of SS in a middle-aged female with a past medical history of dementia.

## Case presentation

A 54-year-old female with a past medical history of dementia was brought to the emergency department (ED) from a nursing home for diminished sensorium and agitation that had started three hours before. Upon arrival, she was irritable, belligerent, impulsive, and unable to be redirected. She tried to attack the staff. The patient had to be chemically sedated in the ED with the help of diphenhydramine 50 mg, lorazepam 2 mg, and haloperidol 5 mg all given intramuscularly. Later, she was somnolent, not responding to commands, and not oriented to time, place, and person. Her vitals were pulse rate - 74/min, blood pressure (BP) - 138/90 mm Hg, temperature - 98.6℉, and respiratory rate of 18/min on arrival. Family history and social history could not be obtained. CT brain without contrast did not show any significant abnormalities. CT cervical spine was also unremarkable. The urine drug screen was negative and serum ethanol levels were less than 10 mg/dL. Her initial labs were significant for elevated creatine phosphokinase (CPK) (753 U/L). Her muscle tone and reflexes were normal. The patient was admitted for further investigations and management. A psychiatric consult was sought, stating that the patient would have had a long-standing history of poorly controlled underlying bipolar disorder and that the current episode could be a mixed manic depressive episode. Her family said her home medications were trazodone 150 mg PO OD, sertraline 50 mg PO OD, lorazepam 0.5 mg PO every six hours pro re nata (PRN), memantine 10 mg PO OD, and donepezil 10 mg PO quaque hora somni (QHS) which she had been taking for an unknown duration. IV fluids were started for elevated CPK. The patient was also started on quetiapine 100 mg PO BID for underlying bipolar disorder, and other home medications were continued.

On the first day of admission, the patient appeared drowsy and hypoactive. She was curled up on her bed and refused to take her medications. On her second day of admission, the patient appeared restless, and her speech was slurred. She had insomnia and diaphoresis. She could also be seen crying which could have been due to pain. Her blood pressure was elevated to 147/91 mmHg, and other vitals were normal (pulse rate - 96/min, temperature - 97.7℉, respiratory rate - 18/minute, oxygen saturation - 97%). Her central nervous system examination was significant for increased muscle tone in all four extremities, bilateral brisk reflexes, and a positive Babinski’s sign. Intermittent low amplitude myoclonic jerks, ocular clonus, and mild intermittent tremors could be seen. A complete blood count, comprehensive metabolic panel, thyroid function tests, erythrocyte sedimentation rate, serum vitamin B12, iron and folate levels, rheumatoid factor, and the antinuclear antibody titer was ordered. All her labs were within reference range except for increasing CPK levels - from 753 U/L which increased to 1973 U/L and to 2395 U/L on the third day of admission. SS was diagnosed using Hunter’s criteria. Her serotonergic medications - sertraline 50 mg OD and quetiapine 100 mg BID were stopped. Lorazepam dose was increased from 2 mg Q8H to Q6H. IV fluids were continued at the rate of 100 mL/h. The diaphoresis and restlessness improved and the patient started calming down after the serotonergic agents were stopped. However, hyperreflexia and clonus were persistent. On the third day of admission, cyproheptadine was started, with a bolus of 12 mg PO given through a nasogastric tube. She was later given a maintenance dose of 8 mg PO every 6 h.

By the fourth day of admission, the patient’s mood was stable. She had mild hyperreflexia and mild tremors. She started making eye contact. Her serum CPK levels started trending down. On the day of discharge, the patient was tolerating oral feeds well and she was able to feed herself. Lorazepam 2 mg Q6H and cyproheptadine 8 mg PO Q6H were stopped. Her tremors and hyperreflexia markedly improved. She could move all four extremities, and walk with the help of a walker. Further plans of care for dementia and bipolar disorder were discussed with her primary care physician (PCP) and psychiatrist. She was transferred back to the nursing home.

## Discussion

Epidemiology

The accurate incidence of SS is not known due to many reasons: being a relatively rare condition that is not easy to identify in a randomized clinical trial, and it goes undetected and under-diagnosed [1]. It is noted across a broad range of ages from neonates to the elderly and the increasing occurrence is likely due to an increased clinical usage of serotonergic drugs [2]. A point of special interest is that about half of the cases of SS reported to the U.S. Food and Drug Administration (FDA) Adverse Event Reporting System (FAERS) is a single drug which is possibly surprising due to the usual impression of a multi-drug etiology [7].

Risk factors and causative agents

Serotonin syndrome is presumed to be caused by excessive stimulation of serotonin receptors. Animal studies and a small number of investigations done in humans appear to confirm this theory, however, the pathophysiology of SS is probably more complicated, implying other neurotransmitters, and is not fully understood [3]. The drug classes involved in SS can be classified into serotonin precursors, inhibitors of serotonin metabolism, inhibitors of serotonin reuptake from the synaptic cleft, direct serotonin receptor agonists, and drugs sensitizing serotonin receptors [1]. SS is caused by antibiotics, analgesics, antiemetics, antidepressants, antimigraine medications, herbal supplements, drugs of abuse, over-the-counter medications, monoamine oxidase inhibitors, and weight reduction agents [2-3].

Pathophysiology

Serotonin or 5-hydroxy tryptamine or 5-HT is a monoamine neurotransmitter mostly synthesized by enterochromaffin cells of the gastrointestinal tract. They are located in the Raphe nuclei in central nervous system (CNS) and are involved in the regulation of mood, sleep, pain, thermoregulation, and emesis. In the peripheral nervous system, serotonin is involved in many additional functions including vasoconstriction, uterine contraction, bronchoconstriction, gastrointestinal motility, and coagulation [8]. There are at least seven families of 5-HT receptors with subtypes for each. SS results from the agonism or antagonism of various combinations of 5-HT receptor subtypes. 5-HT1A receptor agonists have a role in some effects like hyperactivity and anxiety. Life-threatening effects including hyperthermia and hypertonicity are primarily mediated by 5-HT2A receptor antagonists, which require higher concentrations of serotonin [9].

Clinical presentation

Since the diagnosis of SS is merely done clinically, a comprehensive history and thorough physical and neurological examinations are key. A highly variable presentation is seen, varying from mild to a potentially fatal syndrome. Generally, the onset of symptoms is seen within 24 h of consuming the causative agent/agents in SS, unlike the onset of neuroleptic malignant syndrome, and most of these patients seek medical care within 6 h ( the onset of symptoms was within 6 h for 61.5% of these patients and 25.6% presented after 24 h). Nevertheless, these claims are based on a review of 41 cases by Mason et al. from 1995 to 1999 [8], and in a meta-analysis that was more recently done, they found that only 27.5% of cases had a time of onset within 6 h and 44.5% presented after 24 h [10]. Therefore, these cases can occur within a varied time frame based on the context within which SS happens and pharmacokinetic properties, for example, linezolid-associated SS onset is delayed in the elderly [1]. A triad of clinical features are seen in SS-altered mental status, autonomic dysfunction, and neuromuscular excitation, however, the clinical features of this syndrome are rather variable, and not every patient presents with this triad of features. Mild to moderate cases of SS can present with diaphoresis, anxiety, and tachycardia. Severe cases can develop acidosis, disseminated intravascular coagulation, coma, hepatitis, rhabdomyolysis, hyperthermia, and seizures. An immediate progression can be seen from mild to severe symptoms commonly. A good deal of SS cases will resolve within 24 h, but recovery might take three days or longer. Fatalities are not common, and monoamine oxidase inhibitors are usually involved in the more severe cases [11].

Diagnosis

**Figure 1:**
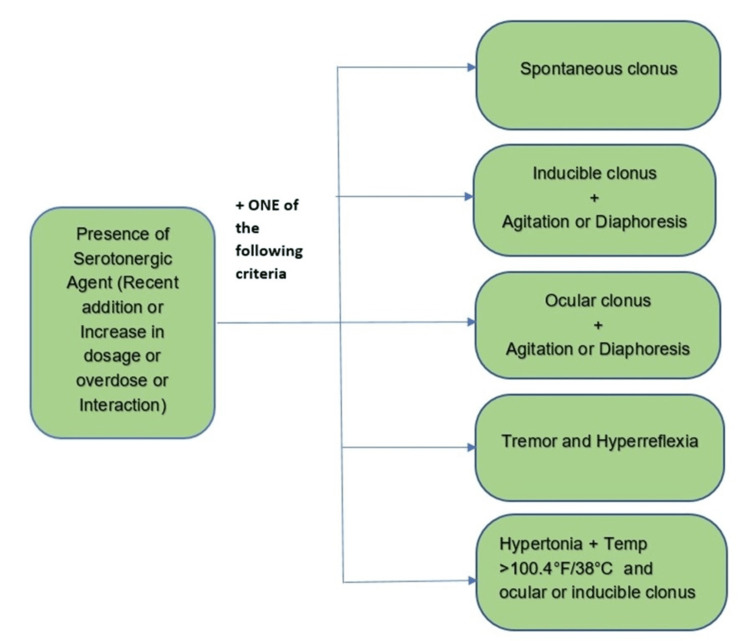
Hunter's criteria for diagnosis of SS. SS, serotonin syndrome

Many diagnostic algorithms have been put forward as SS was first acknowledged as a separate disease entity [11]. Sternbach (SC), Radomski (RC), and Hunter Serotonin Toxicity Criteria (HSTC) are some diagnostic classification systems that are accessible, whereas the most commonly used and generally considered as the gold standard diagnostic criteria is the Hunter Serotonin Toxicity Criteria which focuses on the definitive features of generalized clonus (spontaneous, inducible, and ocular), diaphoresis, agitation, tremor, and hyperreflexia [1, 11]. Hunter's diagnostic criteria are demonstrated (Figure 1).

Differential diagnoses

Serotonin syndrome is a diagnosis of exclusion. Hence considering differential diagnoses is important. The patient was started on haloperidol and quetiapine in the ED, and also had elevated CPK which leads to the possible differential diagnosis of the neuroleptic malignant syndrome (NMS). The features common to both are altered mentation, autonomic disturbances, and neuromuscular hyperexcitability [12]. The differentiating features are the presence of hyperreflexia, muscle rigidity, and ocular clonus in SS whereas hyporeflexia, lead pipe rigidity, and bradykinesia in NMS [2]. SS usually starts rapidly (within minutes to hours) of drug initiation and resolves within 24 h of stopping the offending agent [13-14]. On the contrary, the onset of NMS is slower and can take around 9-14 days to remit despite stopping the offending agents and appropriate treatment [15]. Additionally, mydriasis and hyperactive bowel sounds may occur in SS. The differentiating features of SS and NMS are summarized (Table 1).

**Table 1:** Differentiating features of SS and neuroleptic malignant syndrome. SS, serotonin syndrome

	Serotonin syndrome	Neuroleptic malignant syndrome
Inciting drug	Serotonergic agent	Dopamine antagonist
Onset	Usually < 24 h	Gradual onset (days to weeks)
Vitals	Hypertension, tachycardia, tachypnea, hyperthermia (<41℃)	Hypertension, tachycardia, tachypnea, hyperthermia (>41℃)
Core symptoms	Agitation, diarrhea, diaphoresis, tremors	Dysphagia, hypersalivation
Neuromuscular signs	Hyperreflexia, clonus, mydriasis	Bradykinesia, hyporeflexia, lead-pipe rigidity
Lab findings	Most commonly no significant lab abnormalities	Increase in creatine kinase, leukocytosis, low serum iron

The differential diagnoses of SS other than NMS could be ruled out in this patient because of negative lab evidence (Table 2). Another differential diagnosis that was considered in this patient was familial adult-onset leukodystrophy because of her early-onset dementia and hyperreflexia [16]. But due to a lack of adequate family history and the disappearance of hyperreflexia and clonus with the management of SS, this differential was ruled out.

**Table 2:** Differential diagnoses of SS. SS, serotonin syndrome

Conditions
1. Neuroleptic malignant syndrome
2. Malignant hyperthermia
3. Anticholinergic and sympathomimetic toxicity
4. Thyrotoxicosis
5. Meningitis
6. Encephalitis
7. Heat stroke
8. Sedative-hypnotic withdrawal

Management

Most cases of SS resolve within 24-72 h of discontinuation of serotonergic agents. The key management principles of SS include discontinuation of serotonergic and other neurotropic agents, stabilization of vital signs, maintaining normal oxygen saturation, sedation using benzodiazepines, administering IV fluids, continuous cardiac monitoring, and if refractive, administering serotonin antagonists. Benzodiazepines help in sedating the patient and also correct mild hypertension and tachycardia [2]. For resistant cases, as in our patient, where hyperreflexia and clonus persisted after treatment with benzodiazepines, a non-specific serotonin receptor antagonist like cyproheptadine can be considered. Cyproheptadine has not been shown to reduce the course of SS, but it helps in reducing the symptoms [13, 17]. Antipyretics are not effective for the treatment of hyperthermia in SS. For temperatures greater than 41.1℃, the patient should be transferred to the ICU, sedated, paralyzed using a non-depolarizing agent, and intubated [13]. Medications used in the management of SS can be summarized in Table 3.

**Table 3:** Drugs used in the management of SS and their doses. SS, serotonin syndrome

Drug class		
Sedatives and anticonvulsants	Lorazepam - 2-4 mg intravenously diazepam - 5-10 mg intravenously depending upon the patient response, the doses can be repeated every 8 -10 min.	
Antihypertensives	Esmolol - loading dose of 0.25-0.5 mg/kg IV over 1 min followed by a maintenance dose of 0.05 mg/kg/min for 4 min. Dose can be increased every 10 min by 0.025-0.05 mg/kg/min up to a maximum of 0.2 mg/kg/min. Sodium nitroprusside - 0.25 mcg/kg/min IV infusion. The dose can be titrated every 5 min by 0.25 mcg/kg/min up to a maximum dose of 8 mcg/kg/min.	
Serotonin antagonists	Cyproheptadine - per oral - initial dose of 12 mg followed by 2 mg every 2 h if the symptoms persist. If the patient is stabilized, a maintenance dose of 8 mg every 6 h should be given. Chlorpromazine - 50-100 mg intramuscularly. Not recommended in cases of hypotension, as it can precipitate shock. It can also increase hyperthermia.	
Neuromuscular blocking agents	Rocuronium - 0.01-0.012 mg/kg/min IV vecuronium - 0.01-mg/kg IV to be given 20-45 min after rapid sequence intubation, every 12-15 min as needed to maintain paralysis.	

Complications

A systematic review conducted by Prakash et al. concluded that cardiac complications are the most common in SS and the most frequent reason for fatality [18]. Cardiopulmonary arrest was the most common among cardiac complications, the others being arrhythmia, left ventricular dysfunction, and circulatory collapse. A case of stress-induced cardiomyopathy with a reverse Takotsubo profile following SS has also been reported [19]. Hyperpyrexia and clonus can also precipitate other complications like rhabdomyolysis, myoglobinuria, and acute renal failure [18].

## Conclusions

Serotonin syndrome is a potentially fatal condition. Therefore, it is important to diagnose SS in a timely manner to prevent complications. Our patient was taking sertraline and trazodone. On admission, she has prescribed quetiapine for her suspected poorly controlled bipolar disorder which could have precipitated SS. Stopping all serotonergic drugs promptly is important. Supportive care and cyproheptadine in resistant cases is the treatment strategy. In patients taking serotonergic drugs, caution should be exercised while prescribing drugs that may alter serotonin metabolism. Other differentials such as neuroleptic malignant syndrome should be kept in mind while diagnosing and managing SS.
